# Vaccine Strategies to Improve Anti-cancer Cellular Immune Responses

**DOI:** 10.3389/fimmu.2019.00008

**Published:** 2019-01-22

**Authors:** Karim Vermaelen

**Affiliations:** Tumor Immunology Laboratory, Department of Pulmonary Medicine and Immuno-Oncology Network Ghent, Ghent University Hospital, Ghent, Belgium

**Keywords:** cancer vaccine, adjuvant, dendritic cell, TLR, STING, checkpoint

## Abstract

More than many other fields in medicine, cancer vaccine development has been plagued by a wide gap between the massive amounts of highly encouraging preclinical data on one hand, and the disappointing clinical results on the other. It is clear now that traditional approaches from the infectious diseases' vaccine field cannot be borrowed as such to treat cancer. This review highlights some of the strategies developed to improve vaccine formulations for oncology, including research into more powerful or “smarter” adjuvants to elicit anti-tumoral cellular immune responses. As an illustration of the difficulties in translating smart preclinical strategies into real benefit for the cancer patient, the difficult road of vaccine development in lung cancer is given as example. Finally, an outline is provided of the combinatorial strategies that leverage the increasing knowledge on tumor-associated immune suppressive networks. Indeed, combining with drugs that target the dominant immunosuppressive pathway in a given tumor promises to unlock the true power of cancer vaccines and potentially offer long-term protection from disease relapse.

## Introduction

The aim of a vaccine is to induce an *in vivo* adaptive immune response against a defined antigen or set of antigens. This implies leveraging specific functions of professional antigen-presenting cells in order to trigger T-helper cell responses to support production of antibody production and induce cytotoxic effector T-cells.

The remarkable clinical responses observed with immune checkpoint inhibitors and CAR-T cell therapy have put a definitive end to the discussion whether the human immune system, and T-cells in particular, is capable of controlling or even eradicating cancer. The problem is that vaccination approaches have largely been successful when it comes to inducing humoral immunity, while no major breakthrough has been reached in diseases where cellular responses are also required, such as tuberculosis, HIV, or cancer. For cancer, the bar is raised even higher as vaccines are primarily developed in a therapeutic setting, i.e., with the aim of controlling clinically evident or, at best, minimally residual disease.

The purpose of this review is not to give an exhaustive account of all attempts at cancer vaccination so far, but to provide the reader with the necessary concepts to understand where the field is going, specifically focusing on strategies to elicit clinically meaningful cellular immune responses. Finally, this review will give a perspective of potential combinatorial strategies that could unlock the unique power of vaccines in cancer.

In order for vaccination to deliver unequivocal clinical benefit for cancer patients, improvements must be achieved at two levels: (1) maximizing the induction of a T-cell response with optimal amplitude, specificity and effector profile, (2) ensuring that vaccine-induced T-cells can reach the tumor site and perform their function without any restraint.

The first level involves optimization of the choice of antigenic target(s), of adjuvant potency, and of delivery system. The main principles and some representative preclinical examples in this field will be highlighted in the following section, followed by clinical data (“reality check”) using lung cancer as an illustrative case. In a last section we will outline combinatorial strategies that could herald a revival of cancer vaccines. Molecular formulation of antigens and specific antigen delivery systems constitute a wide domain on their own and will not be handled in detail in this review.

## Optimizing Antigenic Targets

The antigenic landscape in cancer is far more complex than that of viral or bacterial pathogens, where adaptive immunity to well-defined epitopes can drive long term disease protection. In cancer vaccines, it seems rational to target the broadest repertoire of antigens possible in order to avoid selection of escape variants. Approaches that can address this need are the use of autologous tumor lysates, whole tumor-derived mRNA, irradiated autologous tumor cells or allogeneic tumor cell lines (3, 4). All of these pose challenges in terms of logistics, standardization and compliance to regulatory demands including Good Manufacturing Practice (GMP) requirements. Many efforts have been devoted in developing vaccines targeting one or a restricted set of cancer antigens. These can be either differentiation antigens (e.g., MelanA, gp100, tyrosinase), cancer-testis antigens (e.g., MAGE/LAGE/XAGE family, NY-ESO1), or virus-derived antigens (e.g., HPV or EBV-derived proteins) (5). On one hand, this is motivated by practical considerations, including simplicity of vaccine manufacturing and monitoring of immune responses. On the other hand, it is anticipated that effective responses to one antigen, through tumor cell destruction, can lead to an immunogenic release of additional endogenous antigens and spark a broader immune response, a phenomenon known as “epitope spreading” (6).

**Mutanome-derived epitopes** are the most recent addition to defined tumor antigens for use in cancer vaccines. The idea originates from the observation that objective responses to immune checkpoint blockade are proportional to the mutational burden of a given tumor, a number which is the highest in carcinogen-induced cancers (7). This is why the top targets for immune checkpoint inhibition are melanoma, lung cancer and bladder cancer, along with tumors with DNA mismatch repair defects (8). It is now thought that among the total bulk of non-synonymous mutations, a subset that is clonally distributed within the tumor gives rise to mutation-containing peptides (neo-epitopes) that can be recognized by cytotoxic T-cells (9). In addition to single-nucleotide variants, indels have been shown to be strongly predictive of response to immune checkpoint inhibition as well (10). Complex bioinformatic pipelines have been developed to extract a list of candidate immunogenic neo-epitope for a given patient's cancer. This requires deep genomic sequencing of a tumor sample to list all single nucleotide variations (SNVs) and indels. In parallel, RNA sequencing on the same material allows to narrow down on the genomic aberrations that are effectively expressed. Next, *in silico* algorithms are called into action to predict which of the mutations will be presented to T-cells based on proteasome processing and binding affinity for human leucocyte antigen (HLA) molecules. The resulting coding sequences can be synthesized either as peptides as synthetic mRNA. This methodology has been validated in preclinical experiments, showing that vaccination with mutanome-derived neo-antigens can induce protective and therapeutic immune response to autologous tumors (11). Today, this ambitious approach, entirely patient-individualized has entered clinical development with recent phase 1 data demonstrating the feasibility, safety and immunogenicity of neo-antigen-targeted vaccine in metastatic melanoma (12). Notwithstanding the sophistication of this approach, two concerns can be brought forward: (1) several algorithms exist for the prediction of neo-epitopes, and the list of candidate antigens produced for a given tumor can be influenced by the bioinformatic pipeline used, (2) the whole process from next-generation sequencing until manufacturing and release of a GMP-compliant mutanome-derived mRNA vaccine currently takes around 100 days (12), implying that only patients with maximally debulked or relatively indolent tumors are optimally eligible.

## The (Very Crowded) Road TOWARD Optimal Cancer Vaccine Adjuvants

The benefit of adjuvants are best described by the operational definition of Gaston Ramon, better known as the father of the diphtheria vaccine (13): “substances used in combination with a specific antigen that produce more immunity than the antigen alone.” Finding adjuvant formulations that can unlock clinically relevant immune responses against cancer antigens has remained a challenging task: for one, cancer antigens are often poorly immunogenic due to partial homology with self-antigens; on top of that, the optimal cancer vaccine adjuvant must succeed in driving a type 1-polarized, cell-mediated immunity rather than a type 2-polarized and/or humoral response.

Adjuvants can be subdivided in two major classes: (1) immunostimulatory molecules that trigger innate immune receptors, and (2) particulate adjuvants which mainly act either as antigen depots or as delivery systems. Immunostimulatory adjuvants mostly consist of molecules that mimic pathogen-associated molecular patterns and engage Toll-like receptors (TLRs) on antigen presenting cells (APCs) including B-cells, macrophages and most importantly dendritic cells (DCs). In the case of DCs this results in a complex and highly coordinated cellular response aimed at sparking adaptive immunity: (1) switch from antigen uptake mode to antigen processing and presentation, upregulation of a whole array of T-cell costimulatory molecules, upregulation of chemokine receptors mediating migration into T-cell areas of draining lymphoid tissues, and release of specific cytokines and chemokines to polarize the resulting T-cell response. Due to their immunostimulatory power and the capacity to prime naïve T-cells, properly activated DCs are also referred to as “nature's adjuvants.” The use of *ex vivo*-generated and antigen-loaded **DCs as cellular vaccines** will be reviewed in a different article of this Special Edition. The following paragraphs provide a non-exhaustive overview of some of the most notable acellular adjuvant systems optimized for use in cancer vaccines.

### Immunostimulatory Adjuvants: TLR Ligands and Beyond

Among **immunostimulatory adjuvants**, TLR4 ligands constitute some of the most potent members in terms of APC activation. Lipopolysaccharide (LPS), the prototype TLR4-ligand, cannot be used as such in clinical formulations due to toxicity issues. MPL (3-O-desacyl-4′-monophosphoryl lipid A) is a chemically detoxified form of LPS derived from strain R595 of *Salmonella minnesota*, while still retaining immunostimulatory properties (14). It is the only defined TLR ligand approved as part of a vaccine in humans to this day and is a key ingredient of the AS04 adjuvant formulation used in the commercially available HPV and HBV vaccines. However, what makes MPL especially attractive with respect to anti-cancer vaccination is its capacity to induce robust Th1-polarized and cell-mediated immunity. MPL is also an ingredient of the DETOX adjuvant system, when combined with cell wall peptidoglycans from Mycobacteria (15). DETOX is the adjuvant used in the Melacine^®^ vaccine formulation, which incorporates lysate from two allogeneic melanoma cell lines and has shown some modest clinical benefit in resected stage III melanoma patients (16). Likewise, CG-enriched oligodeoxynucleotides (CpG), by triggering the intracellular TLR9, have also been described as powerful inducers of Th1 and cytolytic T-cell responses. These properties have led the incorporation of MPL together with CpG as part of the proprietary adjuvant formula AS15 in the MAGE-A3-targeted cancer vaccine developed by GSK Biologicals (17). Because of biosynthetic variability in the structure of bacterial-derived LPS and downstream hydrolytic steps, MPL is a heterogenous mix of closely related structures (“congeners”). Hence, synthetic TLR4 agonists have been designed, i.e., aminoalkyl glucosaminide 4-phosphates (AGPs) such as glucopyranosyl lipid A and RC-529 (18). The latter has shown its capacity to induce Th1 responses equivalent to MPL, and still with much lesser *in vivo* toxicity than LPS (19). Several other extra- and intracellular TLR-ligands have been the subject of intensive research efforts [reviewed in (20)], and all have shown value to varying degrees in diverse preclinical tumor models. Although some molecules such as the TLR7/8 agonist imiquimod or the TLR2/4-stimulating preparation Bacille-Calmette-Guérin (BCG) are used routinely in the clinic as standalone therapies, no TLR agonist has so far successfully entered standard of care as an ingredient of a cancer vaccine.

It should be noted that triggering TLR signaling also activates homeostatic counterregulatory mechanisms. These include release of IL-10 by myeloid cells, induction of regulatory T-cells (Tr1), and upregulation of the T-cell checkpoint molecule programmed death ligand-1 (PD-L1) on APCs: all of which contribute to the further induction of T-regs and the dampening of anti-tumor cellular immune responses [reviewed in (21)]. The TLR ligands Pam2Cys (TLR2), LPS (TLR4), imiquimod (TLR7) and CpG (TLR9) all induce IL-10 production, and blockade of IL-10/IL10R axis in these settings augments immune responses (17, 18) Similarly, the TLR3-ligand poly I:C induces PD-L1 on DCs, while PD-L1 blockade boosts effector CD8+ T-cell expansion after a tumor vaccine involving poly I:C as adjuvant (22). Another counterregulatory mechanism after TLR stimulation is the upregulation of indoleamine 2,3-dioxigenase expression in DCs, a side-effect observed with CpG oligodeoxynucleotides (23). IDO is a well-described mediator of immunological tolerance: by depleting tryptophan and generating toxic catabolites, IDO enzymatic activity suppresses T-cell activation and promotes T-reg induction in the tumor micro-environment (discussed in more detail below).

A different class of immunostimulatory adjuvants does not belong to bacterial or viral pathogen-associated molecules but consists of extracts from plant origin. **Saponins** derived from the bark of the South American soapbark tree (*Quillaja saponaria*) contain a family of water-soluble, structurally diverse molecules with strongly pro-inflammatory properties. **QS21** is one of the RP-HPLC fractions of *Q. saponaria* extracts that has been used the most in vaccine development (24). The triterpene aldehyde group is considered as the adjuvant active site, resulting in preclinical models in a strong mixed T-helper 1 (Th1), CD8 T-cell and humoral response. QS21 was shown to primarily activate the ASC/NALP3 inflammasome pathway, which converts pro-IL-1β and pro-IL-18 into their bioactive forms (25). This provides the rationale to combine with a TLR4 ligand in order to induce upstream expression of the pro-forms. Still, it appears that the magnitude and quality of the resulting immune response is not proportional to the degree of inflammasome activation, and high doses of QS21 can cause cell membrane lysis and apoptosis of APCs (25). QS21 has been tested extensively in therapeutic cancer vaccine formulations involving ganglioside antigens (GD2, GD3, or GM2) (24). Although robust and humoral responses were invariably observed, there was no convincing evidence of cell-mediated immunity in humans. QS21 is also combined with MPL as part of the AS01 and AS15 adjuvant formulation (GSK), as evaluated in the MAGE-A3 cancer vaccines (discussed below).

**STING** agonists are a recent addition to the arsenal of candidate vaccine adjuvants. STING (STimulator of INterferon Genes) is a transmembrane protein located in the endoplasmic reticulum that belongs to the family of nucleic acid sensors (26). STING activation triggers robust type 1 IFN responses in a TBK1-IRF3-dependent way as well as IKK/NFkB-dependent upregulation of inflammatory cytokines and chemokines. STING can be activated in two ways. The presence of cytosolic double-stranded DNA (e.g., originating from invading DNA viruses or self-DNA from stressed/damaged cells) is first detected by the cGAS molecule which generates cyclic 2′3′-GMP-AMP (cGAMP) from ATP and GTP. As a second messenger, 2′3′-cGAMP then goes on to bind and activate STING, triggering both IRF3- and NFkB-dependent immune/inflammatory gene expression. cGAS expression is by itself inducible by type I interferon, which provides a positive feedback mechanism when relevant ligands persist. Alternatively, STING can be directly triggered by bacterial cyclic dinucleotides such as c-di-GMP. In preclinical models, high doses of c-di-GMP injected intratumorally can directly induce caspase 3-dependent apoptosis of tumor cells and release of tumor-associated antigens, while lower exposure to c-di-GMP can lead to activation of DCs and promote CD8+ T-cell responses against those antigens (27). Other preclinical studies have demonstrated the value of STING agonists in the setting of therapeutic cancer vaccination (28). Caution must be paid however as among immune cells, STING expression is the highest in T lymphocytes. STING activation has been shown to lead to T-cell apoptosis, a phenomenon that appeared cell-specific as macrophages and DCs did not display such sensitivity (29). Hence, implementation of STING agonists in cancer vaccines should ideally be combined with adjuvant/antigen delivery systems that specifically target myeloid cells *in vivo*, as already reported (30). A potential bonus with this type of approach is that STING agonists can reprogram myeloid-derived suppressor cells toward a DC-like immune-stimulating phenotype expressing IL-12 and T-cell costimulatory molecules (27). Another difficulty in translating preclinical data to clinical development strategies is the fact that STING agonists can have differential binding properties in murine vs. human cells. The flavonoid compound DMXAA for instance can bind mouse STING and induced anti-tumor immunity, but fails to activate human STING (31). Still, based on its unique properties, the STING pathway has become a “hot” candidate in the pipeline of several biotech and larger pharmaceutical companies (IFM Therapeutics, Selvita, iTeos, MSD). To date few compounds have reached the stage of early clinical development: ADU-S100 (Novartis) and MK-1454 (MSD). Due to systemic toxicity, both require accessible lesions for intratumoral injection, and both are (quite rationally) combined with systemic administration of an immune checkpoint inhibitor (NCT03172936, NCT03010176).

Next to pathogen-derived molecules, specific host proteins have been shown to perform adjuvant-like functions as well. Immunostimulatory cytokines such as IL-2, IFN-γ, IL-12 and **granulocyte-macrophage colony stimulating factor** (**GM-CSF**) represent an obvious choice as an ingredient for a vaccine. By far the most used in clinical trials is GM-CSF. Based on preclinical studies, GM-CSF helps in the recruitment of dendritic cells to the vaccine injection site, promotes DC maturation and antigen-presentation, resulting in enhanced adaptive immune responses (32). GM-CSF is also the essential ingredient for the *ex vivo* generation of monocyte-derived DCs for vaccination purposes, as discussed elsewhere in this edition. GM-CSF has been incorporated in vaccine formulations either as a standalone adjuvant, or in the shape of allogeneic tumor cell lines engineered for stable expression of GM-CSF (GVAX^®^) (32). A concern still persists as to the optimal dosage of GM-CSF however, with preclinical studies indicating the potential of this cytokine to expand MDSCs, with paradoxical suppression of T-cell mediated anti-tumor responses *in vivo* as a consequence (33). This effect on MDSCs was also observed in clinical trials, where a low-dose GM-CSF added to a cancer vaccine caused a systemic expansion of an immunosuppressive CD14-positive HLA-DR-low/-negative myeloid cell subset. In an another controlled clinical trial, including GM-CSF as part of an incomplete Freund's adjuvant formula resulted in significantly lower T-cell responses to vaccine antigens compared to adjuvant *without* GM-CSF (34). Still, a surprisingly large number of trials using GM-CSF as an adjuvant component are active (listed in Supplementary Table); their results will need to be interpreted with caution.

A different class of endogenous proteins with immunogenic activity are **heat-shock proteins** (**HSPs**). HSPs are chaperones that are released from stressed or dying (cancer) cells, with the unique property of binding cell-derived peptides (35). These peptides can be delivered to DCs resulting in cross-presentation and induction of efficient CD8+ T-cell-mediated immunity (36). The transfer of peptides from HSPs to the APC's MHC class I molecules is not passive but requires uptake by the HSP receptor CD91 expressed by the APC and internal processing. The repertoire of peptides bound by the HSPs reflects the antigenic make-up of the cell of origin, a property which can be leveraged to induce a broad T-cell-mediated protective immunity. In addition, HSP carrier molecules by themselves act as innate immune stimuli, triggering essential events in APCs including release of TNF-α, IL-1β, IL-12, GM-CSF, inflammatory chemokines, and upregulation of costimulatory molecules (37). This effect could be due to binding of HSPs to TLR4, which reinforces the notion that HSPs constitute *bona fide* endogenous adjuvants. Immunization with tumor cell-derived HSPs such as HSP70 and GP96 has demonstrated impressive protective immunity in several preclinical studies [reviewed in (38)]. This has led to the clinical development of autologous HSP96-based vaccines formulation (e.g., vitespen / Oncophage^®^). Clinical trials have shown that this therapy is feasible and non-toxic, although clinical benefit was low except maybe in subset analyses including early-stage renal cell cancer (RCC) and a trend toward benefit in M1a/M1b melanoma patients (39, 40). With these results, vitespen failed to obtain approval from the European Medicines Agency (EMA). Also, one major limitation for further development of HSP-based vaccines is the manufacturing process itself which requires access to sufficient amounts of autologous tumor material. Still, a number of combination clinical trials implementing HSP-based vaccines are ongoing (Supplementary Table).

### Particulate Matter Adjuvants

The most widely used particulate adjuvants historically have been aluminum salts, mostly in the shape of aluminum hydroxide (“alum”). Alum triggers innate immune responses in a TLR-independent way but rather stimulates the NALP3 inflammasome. Being very potent in inducing pure T-helper 2 (Th2) and antibody responses, alum salts are by themselves unfit for use in cancer vaccines. However, when associated with type-1 polarizing ingredients such as ISA 51 (Montanide, see below) and recombinant IL-12, alum was shown to enable a more sustained immune response to tumor-associated antigens probably due to a depot / slow release effect (41). Likewise, combining alum with MPL (GSK's AS04 adjuvant formula) enables a more sustained type-1 polarized cytokine response (42). Other particulate adjuvants have been tailored to better respond to the demands of a cancer vaccine (43). The oldest prototype, Freunds adjuvant, is a water-in-oil emulsion containing heat-killed Mycobacteria. Although being very immunogenic in preclinical models, it is much too toxic for human use. A less toxic formulation that incorporates squalene and oleate, **Montanide ISA-51** (“Incomplete Freunds Adjuvant”) has been used in many therapeutic cancer vaccines. This includes a pivotal trial using the melanoma TAA gp100 as target, in which the clinical activity of ipilimumab alone or in combination with a vaccine vs. vaccine alone was assessed in metastatic melanoma patients (44). Despite induction of robust antibody and CTL responses and signals of clinical benefit in small patient cohorts, none of the Montanide-adjuvanted cancer vaccines has reached advanced clinical development in oncology so far. Adjuvants based on oil-in-water emulsions have been subsequently developed and show a superior safety profile, excellent depot properties, but produce strongly Th2-biased and humoral immune responses (15).

It has been observed by many research groups that a key to induce cellular immunity is the capacity to exploit the cross-presentation capacity of dendritic cells. An efficient way to achieve this goal is by packaging antigens in non-soluble particles, such as virosomes, liposomes, ISCOMs, and microspheres (45). **Virosomes** and **virus-like particles** (VLP) are 20–100 nm size and consist of the membrane envelop of a virus (including embedded proteins) but devoid of a replication-competent genome. Nevertheless, VLPs can efficiently fuse with the membrane of the target cell (ideally an APC), simultaneously delivering an antigenic cargo and any PAMP that can be incorporated in the design. A successful VLP-based vaccine is Gardasil^®^, which contains capsid proteins of HPV serotypes 6, 11, 16, and 18. The vaccine uses aluminum hydroxide phosphate sulfate as adjuvant and is hence a potent inducer of long-lasting and very protective humoral immune responses.

Considerable experience has also been gathered with **ISCOMs**, which are 40 nm micellar structures in which a saponin adjuvant (QS21) and protein antigen is incorporated. ISCOMATRIX consists of just the micellar components and adjuvant, with the flexibility of adding an antigen of choice. ISCOMs differ from liposomes as the latter contain an internal aqueous space confined by a lipid bilayer. As a consequence of the built-in saponin, ISCOMs exert their adjuvant activity by activating the NALP3 inflammasome, while delivering antigenic cargo to dendritic cells to cross-prime CD8+ T-cells (46). *In vivo*, tumor antigen-specific cellular and humoral immune responses were observed after vaccination with NY-ESO1-containing ISCOMs (47). Further intensive research efforts are being devoted to engineer **novel synthetic particles** with the aims of maximizing vaccine potency while specifically targeting cross-presenting APCs. The wide spectrum of physico-chemical parameters that can be varied in the manufacturing such next-generation nanoparticles offers great flexibility in terms of targeting and immunostimulatory properties (see (48) for a comprehensive overview).

## Optimizing Cancer Vaccine Formulations: a Reality Check

The solid preclinical rationale upon which several types of vaccine designs are based stands in sharp contrast to the sobering clinical results observed. Here, we summarize vaccine development in non-small cell lung cancer (NSCLC) as a good example of the limited clinical benefit of cancer vaccines as monotherapy. Many of the strategies described in the previous section have been tested clinically in lung cancer, be it protein-, liposome-, VLP-based or genetically engineered whole cell vaccine platforms.

One of the largest clinical trials ever undertaken in NSCLC was a randomized, double-blind, placebo-controlled phase 3 study using GSK Biological's recombinant MAGE-A3 vaccine (49). The formulation contains full-length recombinant MAGE-A3 protein, a cancer-testis antigen expressed in about 40% of NSCLC patients, combined with the AS15 adjuvant system described earlier. Despite the cancer-specificity of MAGE-A3, notwithstanding the strong type-1 polarizing activity of the AS15 adjuvant formulation and promising phase 2 trial data, the phase 3 trial showed no benefit at all in terms of overall and disease-free survival in early-stage NSCLC patients vaccinated after surgical resection (49). Moreover, an “immune-activated” predictive gene expression signature identified in the melanoma MAGE-A3 vaccine trials failed to identify a MAGE-A3+ NSCLC patient subset who might benefit from vaccination. The vaccine produced strong and long-lasting antibody responses, in line with early clinical data (50), but no convincing evidence for the induction of cytotoxic T-cell responses was provided in this trial. In part due to these results, development of a similar vaccine targeting the cancer-testis antigen PRAME in NSCLC was stopped prematurely (51).

L-BLP25 (Stimuvax^®^) is a liposomal formulation incorporating as antigen a synthetic lipopeptide coding for 25 amino acids of the Muc-1 protein (tecemotide), and MPL as adjuvant. Muc-1 is a glycoprotein that is overexpressed and typically aberrantly glycosylated in a several adenocarcinomas, among which a large subset of NSCLC. L-BLP25 failed to demonstrate a benefit in overall survival in the intention to treat population in a phase III trial involving locoregionally advanced NSCLC patients after chemo-radiotherapy (START trial, NCT00409188) (52). However, a major increase in median OS was observed in the subgroup of patients who received concurrent rather than sequential chemoradiotherapy. These results were meant to be verified in a follow-up phase 3 trial (START2, NCT02049151), however based on negative results of a trial in Asian NSCLC patients (INSPIRE, NCT01015443) (53) the sponsor decided to stop development of L-BLP25 (“Stimuvax”) in all indications.

TG4010 is another Muc-1-targeting vaccine evaluated in NSCLC. It consists of a replication-deficient viral vector, modified vaccinia Ankara (MVA), expressing both Muc-1 as well as IL-2 to support T-cell proliferation. In preclinical models, MVA induces expression of the incorporated antigen sequence in target tissues at equivalent levels compared to replication-competent virus, albeit with a faster kinetic (54). MVA can trigger type-1 IFN production in a TLR-independent fashion. This, combined with the induction of not only humoral but also of type-1-polarized cellular immune response makes MVA theoretically an attractive tool for cancer vaccination purposes. A first trial in advanced NSCLC gave indication of benefit when combined with 1st line chemotherapy, vs. chemotherapy + placebo (55). This prompted a confirmatory phase 2b/3 trial that included a candidate predictive biomarker (the percentage of activated NK-cells in peripheral blood). Results of the phase 2b part showed a significant increase in progression-free survival (PFS; primary endpoint) that was most pronounced in non-squamous NSCLC (where Muc-1 expression is expected to be the highest) and with biomarker value in the lower 3 quartiles (56). Results of the phase 3 part are still pending.

As a final example, in an attempt to target a broad repertoire of antigens, a vaccine was designed containing four irradiated NSCLC allogeneic cell lines (belagenpumatucel-L, Lucanix^®^). In addition, the cell lines where genetically engineered to express an antisense gene vector that inhibits TGF-β2 expression. TGF-β2, along with IL-10, is a prototypical mediator of tumor-induced immune suppression and T-reg induction, and introduction of TGF-β2 antisense plasmid was shown to increase vaccine immunogenicity in preclinical studies (57). It must be stressed though that while the production of TGF-β2 by the vaccine cells themselves is suppressed, this does not affect the levels of this suppressive cytokine emanating from the tumor microenvironment. Belagenpumatucel-L has been evaluated as consolidation therapy in locally advanced and metastatic NSCLC patients that had not progressed on their last line of chemotherapy. Data from a phase 2 trial appeared promising with a clear dose-dependent increase in overall survival (58). However, in a follow-up phase 3 study, no benefit in OS was observed except in a subgroup of patients that had received radiation and chemotherapy < 6 months prior to randomization (59). Patient numbers in this subgroup were very small though and to this day it remains unsure whether this analysis will prompt a confirmatory phase 3 study focusing on this subpopulation.

The impossibility or at best difficulty to demonstrate unequivocal clinical benefit in these vaccination trials raises many questions. When it comes to cancer immunotherapy, the avalanche of robust and positive data coming from the immune checkpoint inhibitor field represents today's benchmark. Patient outcomes after vaccination highlight the difficulty of inducing productive cytolytic responses against cancer in humans. It is clear that a careful choice of antigenic target, adjuvant formula and delivery platform are not sufficient to elicit therapeutic or protective immunity against cancer. This warrants more attention to the tumor-associated tolerogenic or immunosuppressed climate that reigns in the cancer patient.

## Unleashing Immune Effector Mechanisms Downstream of Vaccine Action

**Figure 1:**
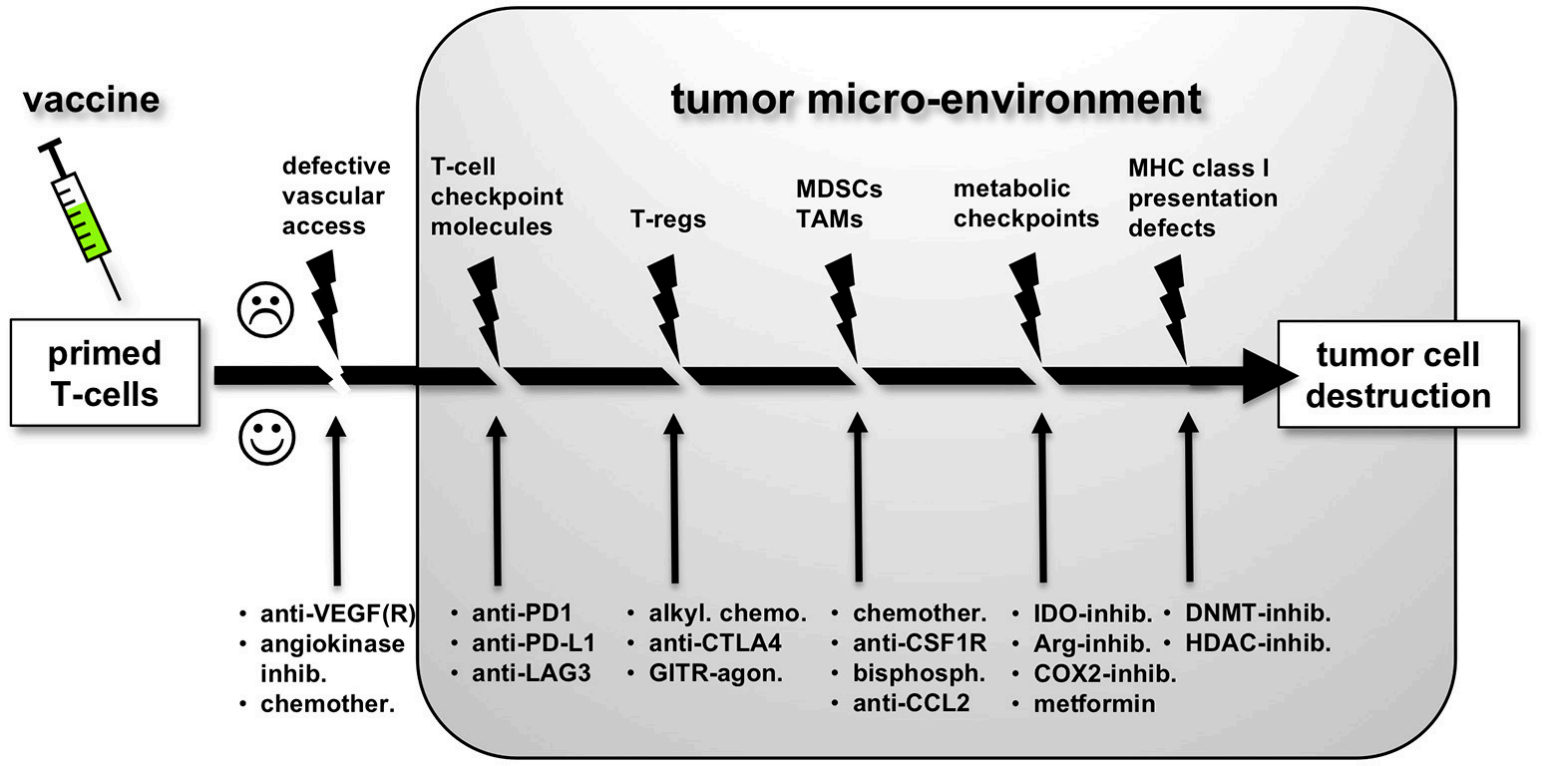
The multiple obstacles to effective anti-tumor immune responses following successful priming of tumor antigen-specific T-cells by a vaccine. Each obstacle offers opportunities for therapeutic intervention in order to increase vaccine efficacy, as discussed in more detail in the main text.

The immune response against cancer cells is a series of critical steps, also described as the “cancer immunity cycle” (60). As a consequence, the strength of the response at the end of this chain of events will be determined by its weakest link (see Figure 1). Each of the obstacles to successful antitumor immune responses have been studied in detail and offers opportunity for therapeutic modulation. Clinical trials exploring combinatorial strategies are summarized in Table 1. The underlying principles will be discussed below.

**Table 1:** Current clinical trial landscape exploring combinatorial approaches to improve therapeutic cancer vaccine efficacy.

**Clinical trial I.D**.	**Study title**	**Interventions**	**Phase**
**(A) Cancer Vaccine + Angiogenesis-Targeting**			
NCT03050814	Standard of Care Alone or in Combination With Ad-CEA Vaccine and Avelumab in People With Previously Untreated Metastatic Colorectal Cancer QUILT-2.004	Drug: Avelumab|Biological: Ad-CEA vaccine|Drug: Bevacizumab|Drug: 5-FU|Drug: Leucovorin|Drug: Oxaliplatin|Drug: Capecitabine	Phase 2
NCT02754362	A Toll-like Receptor Agonist as an Adjuvant to Tumor Associated Antigens (TAA) Mixed With Montanide ISA-51 VG With Bevacizumab for Patients With Recurrent Glioblastoma	Drug: Bevacizumab|Biological: Peptide Vaccine|Drug: Poly-ICLC as immune adjuvant|Drug: Keyhole limpet hemocyanin (KLH)	Phase 2
NCT02432846	Intratumoral Vaccination With Intuvax Pre-nephrectomy Followed by Sunitinib Post-nephrectomy vs. Sunitinib Post-nephrectomy in Newly Diagnosed Metastatic Renal Cell Carcinoma (mRCC)	Biological: Intuvax (ilixadencel)|Drug: Sunitinib	Phase 2
NCT02010606	Phase I Study of a Dendritic Cell Vaccine for Patients With Either Newly Diagnosed or Recurrent Glioblastoma	Biological: Dendritic cell vaccination, in addition to standard temozolomide chemotherapy and involved field radiation therapy|Biological: Dendritic cell vaccination, with optional bevacizumab treatment for patients previously treated with bevacizumab	Phase 1
NCT01814813	Vaccine Therapy With Bevacizumab vs. Bevacizumab Alone in Treating Patients With Recurrent Glioblastoma Multiforme That Can Be Removed by Surgery	Biological: HSPPC-96|Drug: bevacizumab	Phase 2
NCT01551745	Salvage Ovarian FANG Vaccine + Bevacizumab	Biological: Vigil,Ñc Vaccine|Drug: Bevacizumab	Phase 2
NCT01312376	Autologous T-Cells Combined With Autologous OC-DC Vaccine in Ovarian Cancer	Biological: OC-DC vaccine|Drug: Bevacizumab|Drug: cyclophosphamide 300 mg/m^2^/d for 3 days|Drug: fludarabine 30 mg/m^2^/d for 3 days|Drug: *ex vivo* CD3/CD28-costimulated vaccine-primed peripheral blood autologous T cells	Phase 1
NCT01223235	Polyvalent Vaccine-KLH Conjugate + Opt-821 Given in Combination With Bevacizumab	Biological: bevacizumab and the polyvalent vaccine-KLH conjugate + OPT-821	N/A
NCT00913913	Bevacizumab, Autologous Tumor/DC Vaccine, IL-2 and IFNŒ±-2b in Metastatic Renal Cell Carcinoma (RCC) Patients	Biological: DC vaccine|Drug: Bevacizumab|Biological: IL-2|Biological: IFN	Phase 2
NCT00874588	Peptide Vaccine Targeting to Cancer Specific Antigen Combined With Anti-angiogenic Peptide Antigen in Treating Patients With Non-small Cell Lung Cancer	Biological: HLA-A^*^2402restricted URLC10, CDCA1, VEGFR1, and VEGFR2	Phase 1
NCT00828009	BLP25 Liposome Vaccine and Bevacizumab After Chemotherapy and Radiation Therapy in Treating Patients With Newly Diagnosed Stage IIIA or Stage IIIB Non-Small Cell Lung Cancer That Cannot Be Removed by Surgery	Biological: bevacizumab|Biological: emepepimut-S|Drug: carboplatin|Drug: cyclophosphamide|Drug: paclitaxel|Radiation: radiation therapy	Phase 2
**(B) Cancer Vaccine** **+** **TAM/MDSC-Targeting**			
NCT02544880	PDE5 Inhibition Via Tadalafil to Enhance Anti-Tumor Mucin 1 (MUC1) Vaccine Efficacy in Patients With HNSCC	Drug: Tadalafil|Biological: Anti-MUC1 Vaccine|Biological: Anti-Influenza Vaccine|Other: Tadalafil Placebo|Other: Vaccine Placebo|Procedure: Peripheral Blood Collection|Procedure: DTH Skin Test|Procedure: Tumor specimen collection	Phase 1/2
NCT02479230	Type I-Polarized Autologous Dendritic Cell Vaccine With Tumor Blood Vessel Antigen-Derived Peptides in Metastatic Breast Cancer Patients	Biological: tumor blood vessel antigen peptide-pulsed alpha-type-1 polarized dendritic cell vaccine|Drug: gemcitabine hydrochloride	Phase 1
NCT02432378	Intensive Locoregional Chemoimmunotherapy for Recurrent Ovarian Cancer Plus Intranodal DC Vaccines	Biological: Cisplatin + celecoxib + DC vaccine|Biological: Cisplatin + CKM + Celecoxib + DC Vaccine	Phase 1/2
NCT02275039	p53MVA Vaccine and Gemcitabine Hydrochloride in Treating Patients With Recurrent Ovarian Epithelial Cancer	Biological: modified vaccinia virus ankara vaccine expressing p53|Drug: gemcitabine hydrochloride|Other: laboratory biomarker analysis	Phase 1
NCT01876212	Dendritic Cell Vaccines + Dasatinib for Metastatic Melanoma	Biological: DC vaccine|Drug: Dasatinib	Phase 2
NCT01803152	Dendritic Cell Vaccine With or Without Gemcitabine Pre-Treatment for Adults and Children With Sarcoma	Biological: Dendritic Cells Vaccine|Biological: Lysate of Tumor|Drug: Gemcitabine|Drug: Imiquimod|Procedure: Leukapheresis	Phase 1
NCT01697800	A Phase II Trial of Tadalafil in Patients With Squamous Cell Carcinoma of the Upper Aero Digestive Tract	Drug: Tadalafil|Drug: Placebo	Phase 2
NCT02616185	A Phase 1 Study To Evaluate Escalating Doses Of A Vaccine-Based Immunotherapy Regimen For Prostate Cancer (PrCa VBIR)	Biological: PF-06755992|Biological: PF-06755990|Device: TDS-IM Electroporation Device|Biological: Tremelimumab|Drug: Sunitinib|Biological: PF-06801591	Phase 1
NCT02432846	Intratumoral Vaccination With Intuvax Pre-nephrectomy Followed by Sunitinib Post-nephrectomy vs. Sunitinib Post-nephrectomy in Newly Diagnosed Metastatic Renal Cell Carcinoma (mRCC)	Biological: Intuvax (ilixadencel)|Drug: Sunitinib	Phase 2
NCT03153410	Pilot Study With CY, Pembrolizumab, GVAX, and IMC-CS4 (LY3022855) in Patients With Borderline Resectable Adenocarcinoma of the Pancreas	Drug: Cyclophosphamide|Drug: GVAX|Drug: Pembrolizumab|Drug: IMC-CS4	Early Phase 1
NCT02432378	Intensive Locoregional Chemoimmunotherapy for Recurrent Ovarian Cancer Plus Intranodal DC Vaccines	Biological: Cisplatin + celecoxib + DC vaccine|Biological: Cisplatin + CKM + Celecoxib + DC Vaccine	Phase 1/2
**(C) Cancer Vaccine** **+** **T-Reg-Targeting**			
NCT03203005	IMA970A Plus CV8102 in Very Early, Early and Intermediate Stage Hepatocellular Carcinoma Patients	Drug: IMA970A plus CV8102 and Cyclophosphamide	Phase 1/2
NCT03066947	SV-BR-1-GM in Metastatic or Locally Recurrent Breast Cancer	Biological: SV-BR-1-GM|Drug: Cyclophosphamide|Biological: Interferon-alpha-2b	Phase 1/2
NCT02709993	Consolidation Therapy in Patients With Hematologic Malignancies	Biological: TAPA-pulsed DC vaccine	Phase 1/2
NCT02705703	Consolidation Therapy in Patients With Metastatic Solid Malignancies	Biological: TAPA-pulsed DC vaccine	Phase 1/2
NCT02390063	Vaccination in Prostate Cancer (VANCE)	Biological: ChAdOx1.5T4|Biological: MVA.5T4|Drug: Cyclophosphamide	Phase 1
NCT02224599	Treatment of Patients With Progressive and/or Refractory Solid Malignancies	Biological: TAPA-pulsed DC vaccine	Phase 1/2
NCT02223312	Therapy for Progressive and/or Refractory Hematologic Malignancies	Biological: TAPA-pulsed DC vaccine	Phase 1/2
NCT01696877	A Neoadjuvant Study of Androgen Ablation Combined With Cyclophosphamide and GVAX Vaccine for Localized Prostate Cancer	Drug: degarelix acetate|Drug: Cyclophosphamide|Drug: GVAX	Phase 1/2
NCT01192555	Allogeneic Tumor Cell Vaccination With Oral Metronomic Cytoxan in Patients With High-Risk Neuroblastoma	Biological: Neuroblastoma Vaccine (unmodified SKNLP, with gene-modified SJNB-JF-IL2 and SJNB-JF-LTN neuroblastoma cells)|Drug: Cytoxan	Phase 1/2
NCT00703105	Ovarian Dendritic Cell Vaccine Trial	Biological: Ontak DC|Biological: DC vaccination|Drug: Ontak	Phase 2
NCT00626483	Basiliximab in Treating Patients With Newly Diagnosed Glioblastoma Multiforme Undergoing Targeted Immunotherapy and Temozolomide-Caused Lymphopenia	Biological: RNA-loaded dendritic cell vaccine|Drug: basiliximab	Phase 1
NCT00515528	Vaccination Plus Ontak in Patients With Metastatic Melanoma	Drug: 4-peptide melanoma vaccine|Drug: 4-peptide melanoma vaccine plus Ontak|Drug: ontak	Phase 2
**(D) Cancer Vaccine** **+** **Checkpoint inhibition**			
NCT03548467	A Study to Evaluate Safety, Feasibility, Efficacy of Multiple Dosing With VB10.NEO Immunotherapy in Patients With Locally Advanced or Metastatic Cancer	Drug: VB10.NEO	Phase 1/2
NCT03532217	Neoantigen DNA Vaccine in Combination With Nivolumab/Ipilimumab and PROSTVAC in Metastatic Hormone-Sensitive Prostate Cancer	Biological: PROSTVAC-V|Biological: PROSTVAC-F|Drug: Nivolumab|Drug: Ipilimumab|Biological: Neoantigen DNA vaccine|Device: TriGrid Delivery System|Procedure: Tumor biopsy|Procedure: Peripheral blood|Procedure: Fecal samples	Phase 1
NCT03422094	Neoantigen-based Personalized Vaccine Combined With Immune Checkpoint Blockade Therapy in Patients With Newly Diagnosed, Unmethylated Glioblastoma	Biological: NeoVax|Biological: Nivolumab|Biological: Ipilimumab|Procedure: Research blood draw|Procedure: Leukapheresis for research	Phase 1
NCT03362060	PVX-410 Vaccine Plus Pembrolizumab in HLA-A2+ Metastatic Triple Negative Breast Cancer	Drug: Pembrolizumab|Biological: PVX-410	Phase 1
NCT03311334	A Study of DSP-7888 Dosing Emulsion in Combination With Immune Checkpoint Inhibitors in Adult Subjects With Advanced Solid Tumors	Drug: DSP-7888 Dosing Emulsion|Drug: Nivolumab|Drug: Atezolizumab	Phase 1
NCT02654587	Study of OSE2101 vs. Standard Treatment as 2nd or 3rd Line in HLA-A2 Positive Patients With Advanced NSCLC After Failure of Immune Checkpoint Inhibitor	Drug: OSE2101|Drug: Docetaxel|Drug: Pemetrexed	Phase 3
NCT03113487	P53MVA and Pembrolizumab in Treating Patients With Recurrent Ovarian, Primary Peritoneal, or Fallopian Tube Cancer	Other: Laboratory Biomarker Analysis|Biological: Modified Vaccinia Virus Ankara Vaccine Expressing p53|Biological: Pembrolizumab	Phase 2
NCT02977156	Immunization Strategy With Intra-tumoral Injections of Pexa-Vec With Ipilimumab in Metastatic / Advanced Solid Tumors.	Biological: Pexa-Vec|Drug: Ipilimumab	Phase 1
NCT02506114	Neoadjuvant PROSTVAC-VF With or Without Ipilimumab for Prostate Cancer	Biological: PROSTVAC V/F|Drug: Ipilimumab	Phase 2
NCT02432963	Vaccine Therapy and Pembrolizumab in Treating Patients With Solid Tumors That Have Failed Prior Therapy	Other: Laboratory Biomarker Analysis|Biological: Modified Vaccinia Virus Ankara Vaccine Expressing p53|Biological: Pembrolizumab	Phase 1
**(E) Cancer Vaccine** **+** **Costimulation Agonists**			
NCT03258008	Utomilumab and ISA101b Vaccination in Patients With HPV-16-Positive Incurable Oropharyngeal Cancer	Drug: Utomilumab|Biological: ISA101b	Phase 2
NCT01898039	Modified Melanoma Vaccine for High Risk or Low Residual Disease Patients	Biological: A2/4-1BBL melanoma vaccine|Procedure: DNP sensititzation|Drug: Cyclophosphamide	Phase 1/2
NCT01861938	Modified Melanoma Vaccine for High Risk or Low Residual Disease Patients	Biological: Melanoma vaccine modified to express HLA A2/4-1BB ligand	Phase 2/3
NCT01644968	Phase 1 Study of Anti-OX40 in Patients With Advanced Cancer	Drug: Cohort 1 anti-OX40|Drug: Cohort 2 anti-OX40|Drug: Cohort 3 anti-OX40|Biological: Tetanus Day 29|Biological: Tetanus Day 1|Biological: KLH Day 1|Biological: KLH Day 29	Phase 1
NCT00534209	Vaccine Therapy in Patients With Stages IIIB/IV Non-Small Cell Lung Cancer Who Have Finished First-Line Chemotherapy	Biological: Allogeneic B7.1/HLA-A1|Other: Placebo	Phase 1/2
NCT00031564	Phase II Study of a B7-1 Gene-Modified Autologous Tumor Cell Vaccine and Systemic IL-2	Biological: Interleukin-2|Biological: B7-1	Phase 2
**(F) Cancer Vaccine** **+** **IDO-Inhibition**			
NCT02166905	DEC-205/NY-ESO-1 Fusion Protein CDX-1401, Poly ICLC, and IDO1 Inhibitor INCB024360 in Treating Patients With Ovarian, Fallopian Tube, or Primary Peritoneal Cancer in Remission	Biological: DEC-205/NY-ESO-1 Fusion Protein CDX-1401|Drug: Epacadostat|Other: Laboratory Biomarker Analysis|Other: Pharmacological Study|Drug: Poly ICLC	Phase 1/2
NCT03047928	Combination Therapy With Nivolumab and PD-L1/IDO Peptide Vaccine to Patients With Metastatic Melanoma	Drug: Nivolumab|Biological: PD-L1/IDO peptide vaccine	Phase 1/2
**(G) Cancer Vaccine** **+** **Epigenetic Modulation**			
NCT02166905	DEC-205/NY-ESO-1 Fusion Protein CDX-1401, Poly ICLC, and IDO1 Inhibitor INCB024360 in Treating Patients With Ovarian, Fallopian Tube, or Primary Peritoneal Cancer in Remission	Biological: DEC-205/NY-ESO-1 Fusion Protein CDX-1401|Drug: Epacadostat|Other: Laboratory Biomarker Analysis|Other: Pharmacological Study|Drug: Poly ICLC	Phase 1/2
NCT02886065	A Study of PVX-410, a Cancer Vaccine, and Citarinostat +/- Lenalidomide for Smoldering MM	Drug: Hiltonol|Drug: Citarinostat|Drug: Lenalidomide|Biological: PVX-410	Phase 1

*Combinations were structured in line with discussion in the text. Database searches were focused on combinations with agents that target **(A)** angiogenesis, **(B)** MDSCs/TAMs, **(C)** T-regs, **(D)** immune checkpoint molecules, **(E)** costimulatory molecules**, (F)** IDO, and **(G)** epigenetic modifications. No trials were found combining vaccines with interventions targeting immunosuppressive cytokines (IL-10, TGF-β, IL-6), arginase activity, hypoxic metabolism or adenosine signaling. Notes: Database search restricted to clinical trials that are active or will be activated in the near future. Only antigen-specific vaccination protocols were retained (e.g., the use of radiotherapy or intratumoral injections of checkpoint inhibitors was excluded). Combinations with anti-CTLA4 were listed onder “Vaccine + checkpoint inhibition” even though CTLA-4 blockers such as ipilimumab may also directly deplete T-regs. Interventions targeted at hematological malignancies were omitted*.

### Improving Effector T-Cell Access Into the Tumor

Following successful expansion and adequate polarization of tumor-antigen specific T-cells, the latter acquire the capacity of exiting the lymph node and recirculate through the bloodstream to scan for antigens in peripheral tissues. Unfortunately, penetration of effector lymphocytes into tumoral beds is hampered in many ways. Tumor-induced angiogenesis results in a network of aberrant blood vessels in which proper adhesion and extravasation of cytolytic T-cells is impaired. The endothelium of tumoral vasculature is known to be poor in leukocyte adhesion molecules such as intercellular adhesion molecule-1 (ICAM-1) and vascular cell adhesion molecule-1 (VCAM-1). Overactivity of the endothelin-endothelin receptor axis on tumoral endothelia further limits T-cell extravasation by decreasing ICAM-1 expression while further boosting the production of angiogenetic factors such as vascular-endothelial growth factor (VEGF) (61). Similar to physiological immune-privileged organs, the endothelium of tumoral vessels also overexpresses T-cell checkpoint ligands including PD-L1, death receptors such as FasL and TRAIL, and IDO. All of these factors do not seem to hamper the recruitment of T-regs, and together contribute in shielding tumor cells from immune attack. Hence, the clinical benefit obtained with commonly used anti-angiogenic compounds such as the VEGF blocker bevacizumab potentially relies on boosting immune infiltration into tumors (62). Also, inhibition of endothelin receptor signaling has been shown to restore endothelial ICAM-1 expression, increase T-cell infiltration and importantly, act synergistically together with a cancer vaccine (63). Regardless of its prototypical role in angiogenesis, VEGF is also known as a cytokine that suppresses T-cell function and DC activation. Hence VEGF-targeted anti-angiogenic therapy can also exert positive immunomodulatory effects in a cancer immunotherapy setting (64–66).

### Fighting Suppressive Immune Cells in the Tumor Microenvironment

A next obstacle for vaccine elicited T-cells is the influence of several immune suppressive leukocytes that populate the tumor micro-environment, foremost **regulatory T-cells (T-regs)** and **myeloid-derived suppressor cells (MDSCs)**. **T-regs** are known to be preferentially recruited into tumors and inhibit the functions of antitumoral T-cells by producing immunosuppressive mediators such as interleukin-10 (IL-10), transforming growth factor-beta (TGF-β) and adenosine or by consuming interleukin-2 (IL-2) which is critical for cytolytic T-lymphocyte (CTL) proliferation. In a clinical trial involving a NY-ESO1-ISCOMATRIX vaccine in melanoma, absence of clinical efficacy and cellular immune responses was correlated to increased T-reg activity in metastatic compared to early stage patients (67). Preclinical exploration of this phenomenon in a mouse model of pancreatic cancer showed that impaired responses to ISCOM vaccine can be restored by anti-CD25 mAb-mediated depletion of T-regs, or interestingly by adding low-dose CpG-ODN to the ISCOM formulation (68). Numerous other preclinical studies have shown that therapeutic vaccine efficacy can be boosted by depleting T-regs *in vivo* (69). However, selectively eliminating T-regs in a clinical setting is not a straightforward task. As an example the alkylating agent cyclophosphamide can decrease the number of T-regs in cancer patients (70), however this effect is not easily reproducible and is only achieved within a narrow dose range (“metronomic scheduling”). The development of new clinical-grade compounds that can specifically interfere with the suppressive function of T-regs enables interesting combinatorial approaches with vaccines. T-regs typically express high levels of CTLA-4, and the anti-CTLA4 checkpoint inhibitor ipilimumab, being an IgG1-class antibody, can mediate Fc-dependent depletion of these cells in the tumor micro-environment (TME) (71). Glucocorticoid-induced tumor necrosis factor (TNF) receptor related gene (GITR) is another receptor that is highly expressed on T-regs. Engaging GITR with an agonist has the capacity to shut down the immunosuppressive functions of T-regs, while also stimulating CD8+ T-cell function (72). GITR agonists are currently in clinical development as an add-on to anti-PD-1 checkpoint blockade. Preclinical experiments also indicate a clear synergism between GITR agonists and therapeutic cancer vaccines (73, 74), yet to date no clinical trials are investigating this avenue in cancer patients.

**MDSCs** constitute another potential obstacle to vaccine success. This heterogenous population of immature monocytic and granulocytic leukocytes are released from the bone marrow in advanced cancer patients and can severely disrupt CD8+ T-cell function through several mechanisms. For instance, MDSCs produce high levels of nitrogen monoxyde (NO) and reactive oxygen species (ROS), combining to form nitrosamines that impair TCR function (75). MDSCs also typically overexpress arginase 1 which depletes arginine in the TME, thereby depriving effector T-cells with an essential “fuel” for proliferation (76). Tumor-associated macrophages are myeloid cells which share several T-cell suppressive properties with MDSCs. Tumor-associated macrophages (TAMs) release TGF-β, IL-10, pro-fibrogenic, and pro-angiogenetic factors (77).

Several classes of compounds can be “repurposed” to achieve a reduction of MDSCs both systemically and intratumorally, and/or interfere with these cell's suppressive capacity (78). In many cases this results in enhancement of T-cell responses in a therapeutic cancer vaccine setting. This is true for myeloablative **chemotherapeutics** such as platinum salts, taxanes, and anti-metabolites (gemcitabine, 5-FU) (79–81), which are known to decrease systemic MDSC numbers in metastatic cancer patients. In preclinical vaccination models, this has been shown to translate into a boosted in T-cell response to vaccination (82, 83). Alternative strategies to target suppressive myeloid cells include administration of all-trans retinoic acids, triterpenoids, phosphodiesterase inhibitors (e.g., sildenafil), tyrosine kinase inhibitors (e.g., sunitinib), amino-bisphosphonates, recombinant IL-12 and anti-IL-6R monoclonal antibodies (84–89). Anti-CSF-1R and anti-CCL2 can both reduce the recruitment of MDSCs and monocyte-derived TAMs into the tumor bed and also contribute to revert the immunosuppressive climate within tumors (90, 91).

Finally, as noted earlier, next to their adjuvant property in itself, STING agonists have the interesting property of being able to reprogram MDSCs from a T-cell suppressive into a type-1 immune polarizing leukocyte (27).

### Freeing T-Cells From Negative Checkpoint Signals

On a molecular level, tumor beds also maintain a climate of tolerance and immune suppression through the abundant expression of **T-cell checkpoint ligands** and a relative lack of costimulatory molecules. Fortunately, the field of immuno-oncology is currently driven forward by the development of several compounds that can disrupt this inhibitory climate: in a first wave of clinical trials, **immune checkpoint inhibitors (ICIs)** such as **CTLA-4**, **PD-1** and **PD-L1** blocking antibodies have demonstrated unequivocal clinical activity as monotherapy in many types of cancer. The performance plateau of immune checkpoint blockade is now being pushed upward by applying combinatorial strategies (e.g., ICI + chemotherapy or ICI + ICI). It can be expected that combinatorial approaches that include ICIs will be the major development that will unlock the full potential of cancer vaccines. Indeed, a robust activation of T-cells (as potentially achieved by a powerful vaccine) will induce expression of counterregulatory checkpoints such as CTLA-4 and PD-1. CTLA-4 can “steal the steam” of signaling through the B7-CD28 costimulatory axis, hereby shutting down T-cell activation by the APC. PD-1, when engaging PD-L1 which is abundantly expressed on cancer cells and intratumoral myeloid cells by exposure to IFN-γ and/or hypoxia, results in paralysis of T-cell effectors at the tumor front. As a clinical indication for this obstacle to vaccine efficacy, in the trial evaluating the TG4010 Muc-1 vaccine in lung cancer only patients whose tumor expressed low levels of PD-L1 had a marked benefit in progression-free survival (56).

Mechanistically, ICIs can potentiate vaccine responses in two main ways. Anti-CTLA-4 checkpoint inhibition will mainly act by boosting the amplitude of the priming phase, by broadening the repertoire of the T-cell response (92) and also by removing the suppressive activity of T-regs in the TME, as noted earlier. PD-1 or PD-L1 blockade will ensure that vaccine-elicited anti-tumoral T-cells can exert their function unhampered once inside the tumor micro-environment. Conversely, vaccination may be an additional combination partner to improve the performance of checkpoint inhibition, whose response rate as monotherapy across all tumors plateaus around 20% in biomarker-unselected patients.

The benefits of combining vaccines with ICIs have been demonstrated in numerous preclinical tumor models (93–96), and these proof-of-concepts have already led to the design of several clinical trials (summarized in Table 1D). Initial results in humans were not encouraging though, when a pivotal trial showed no benefit at all of combining an adjuvanted gp100 peptide vaccine with anti-CTLA4, compared with anti-CTLA4 alone (44). However, more advanced vaccine platforms may still benefit from combination with ICI, as illustrated by a more recent phase 2 trial exploring the combination of a DC vaccine plus ipilimumab: objective response rates and survival were markedly superior than historical data with ipilimumab as monotherapy (97).

The relative **timing** of vaccination and immune checkpoint blockade could be very critical for optimal anti-tumor effect. CTLA-4 blockade was found to synergize optimally with a prostate cancer GVAX vaccine when administered after vaccination (98). Likewise, responses to TG4010 (Muc-1-targeted MVA vaccine) were enhanced when PD-1 blockade was administered several days after the vaccine (99). By contrast McNeel et al. observed that responses to a PSA-targeted DNA vaccine against prostate cancer were only observed with concurrent rather than sequential PD-1 checkpoint blockade, both in murine models as well as in a small clinical trial (100). The sequencing could be different when it comes to PD-L1 blockade: PD-L1 upregulation is a physiological phenomenon upon DC activation which may serve to protect the DC from elimination during cognate interaction with the CD8+ T-cell. Hence, PD-L1 blockade at the time of vaccination/DC activation may result in abortive T-cell priming due to shortened APC survival and limit effector T-cell polarization and expansion.

Additional checkpoint molecules are currently being explored as clinical targets. Lymphocyte-activation gene 3 (**LAG3)** is the third immune checkpoint to have been targeted in humans after CTLA4 and the PD-1/PD-L1 axis. LAG-3 is expressed by “exhausted” TILs and T-regs. It shares high structural homology to CD4 and binds MHC class II on APCs. Besides keeping the T-cell itself in an inactive state, LAG3 can reverse-signal to the APC and maintain the latter in an immature/pro-tolerogenic state with impaired upregulation of costimulatory molecules and IL-12 secretion (101). LAG3 blockade as such shows limited effects, but it can roughly double the response rate to PD-1 blockade when used in combination, an added benefit that is clearly enhanced in LAG3-expressing tumor beds (NCT01968109, P. Ascierto et al presented at ESMO 2017). Interestingly, a soluble dimeric recombinant protein consisting of four LAG3 extracellular domains fused to the Fc portion of human IgG1 (LAG3-Ig) has been shown to act as an “APC activator” (102). A possible concern however is that it also stimulates release of the chemokines CCL17 and CCL22, which are known to preferentially attract Th2 lymphocytes and T-regs. The clinical compound, IMP321, is now being evaluated in patients in combination with cancer vaccines in different tumor settings (Table 1D).

Besides an abundance in negative checkpoint molecules, the tumor milieu also fosters immune tolerance through a lack in costimulatory molecules. Agonists of T-cell costimulatory pathways are in clinical development, notably monoclonal antibodies that bind to TNF-superfamily receptors such as OX40 and 4-1BB. Preclinical experiments indicate that costimulation agonists can synergize with vaccination to break tolerance toward poorly immunogenic tumors (103, 104), with several clinical trials now underway (Table 1E).

### Dealing With the Immunosuppressive Metabolic Tumor Environment

Next to defined molecular axes, the global metabolic climate within solid tumors provides a hostile environment for proper effector T-cell function as well. An important counterregulatory mechanism in response to an IFN-γ-dominated T-cell attack is the upregulation of **IDO** (indoleamine-2,3-dioxygenase). Also, activation of DCs results in IDO expression in these cells and promotes paradoxical induction of T-regs (105). Prostaglandin E2, generated by COX2-expressing TAMs, is also an inducer of IDO (106, 107). Originally identified as a major contributor to immune tolerance at the maternofoetal interface (108), IDO enzymatic activity is now recognized as one of the “metabolic checkpoints” in tumors such as melanoma and lung cancer: IDO catabolises tryptophan, which is also a “fuel” for proper T-cell activation and proliferation, into kynurenines that act as T-cell toxic metabolites. Tryptophan depletion will also favor the induction of T-regs (109). IDO inhibitors have demonstrated positive effects in many preclinical models of cancer immunotherapy (109). Clinical development of IDO inhibitors took a hit recently with negative phase 3 results in combination with ICI in melanoma, despite promising phase 2 data (NCT02752074, results presented at ASCO 2018). Nevertheless, results in other tumors are still pending, and combining IDO-inhibition with a vaccine may still be an effective strategy (110) (Table 1F). **Arginase** activity is also increased in tumors in proportion to myeloid cell infiltration and induces T-cell paralysis by depleting arginine (as described above). Arginase inhibitors are currently in early clinical development [NCT02903914 (111)], with preclinical data showing clear synergism with anti-PD-L1 checkpoint inhibition (112). No clinical trials combining arginase inhibitors with a cancer vaccine have been reported to date.

More difficult to correct through therapeutic intervention are the consequences of **aberrant energy metabolism** in tumors, where cancer cells out-compete TILs for glucose availability and establish a high lactate/low-pH milieu that blocks T-cell proliferation and IFN-γ release (113). These conditions are further exacerbated by the poor quality of the tumor vasculature which prevents proper clearance of toxic metabolites and exacerbates intratumoral **hypoxia**. The latter induces upregulation of glucose transporters on tumor cells, further decreasing extracellular glucose availability for effector T-cells.

Metformin, better known as a therapy for insulin-resistant diabetes, also inhibits cancer cell oxygen consumption. This has been shown to decrease tumoral hypoxia, hereby augmenting intratumoral CD8+ T-cell activation and unlocking synergistic effects with checkpoint blockade in otherwise immunotherapy-resistant tumors (114).

Hypoxia also increases expression of **ectonucleotidases** on the cell membrane of cancer cells and myeloid cells, resulting in degradation of ATP to **adenosine**. Adenosine triggers A2AR, the most predominant adenosine receptor on immune cells, leading to an increase in intracellular cAMP levels which mediates a plethora of immunosuppressive effects: inhibition T-cell and NK-cell functions, suppression of DC maturation and IL-12 secretion, increase in IL-10 production, induction of T-regs (115).

A2AR antagonists have been developed, with preclinical studies showing promising activity. In a phase I trial the A2AR antagonist CPI-444 produced marked CD8 T-cell infiltration when comparing pre- vs. post-treatment biopsies (116). Preliminary clinical data suggests synergism with PD-L1 blockade, however it is clear from their biological effect that adenosine receptor or ectonucleotidase inhibitors could be attractive add-ons in a therapeutic vaccine setting.

### Improving Tumor Visibility to the Immune System

For vaccine-induced T-cells to fulfil their final role, in addition to intratumoral penetration and surmounting suppressive mechanisms, tumor cells must expose sufficient levels of relevant antigen on their surface. This cannot be taken for granted as cancer cells can reduce expression of tumor-associated antigens or downregulate critical components of the antigen-processing and MHC presentation machinery. Interestingly, this loss of “visibility” to the immune system seems to be mediated by **epigenetic mechanisms**, i.e., DNA hypermethylation and histone deacetylation, which opens up opportunity for therapeutic modulation (117). Expression of cancer-testis antigens is in particular regulated through epigenetic mechanisms, and treatment with DNA methyl transferase (DNMT) inhibitors can increase cancer-testis antigen (CTAG) expression levels on cancer cells. Components of the antigen-processing machinery (APM) such as TAP-1, TAP-2, LMP-2 and Tapasin can be increased by treatment of cancer cells with histone deacetylase (HDAC) inhibitors, which ends up increasing surface expression of MHC class I molecules as well (118, 119).

In addition, epigenetic drugs can help create a more favorite immunological climate within tumors. HDAC inhibitors have been shown to induce Th1, CD8 and NK-cell-attracting chemokines and boost response to anti-PD1 immune checkpoint blockade (120). The combination of DNMT and HDAC-inhibition can also potentiate ICI efficacy by reducing granulocytic MDSC levels (121). Another fascinating discovery is the fact that DNMT-inhibitors can awaken expression of endogenous retroviral vectors (also known as long terminal repeat retro-transposons), thus generating intracellular dsRNAs that can be sensed by the MAD5/MAVS cytosolic sensor and trigger type 1 interferon responses (122).

A large number of clinical trials are now combining checkpoint inhibitors with epigenetic modulators, however only 1 trial exploring the combination a DNMT-inhibitor with a DC-based cancer vaccines in pediatric sarcoma has been completed: remarkably 1 patient of the 10 included experienced a complete response (123). A few other trials combining vaccination with epigenetic modulation are active at the time of this writing (Table 1G).

## Conclusion

Given the daunting complexity of tumor-associated immune suppressive networks, it comes as no surprise that vaccination in a therapeutic setting has delivered so little benefits to cancer patients so far. Still, the overwhelming amount of preclinical data supports the notion that vaccination can control or even eradicate tumors, just as preclinical work showed the value of immune checkpoint blockade many years ago. Given the multiple obstacles to T-cell mediated cancer cell destruction, it is clear that the success of a vaccine will depend on our capacity to accurately map the dominant immunosuppressive pathway for each individual patient. An essential aspect when it comes to therapeutic modulation of these pathways is to delineate the hierarchy of obstacles to effective immune responses. For instance, combining a vaccine with immune checkpoint blockade is an effort in vain when a large part of the tumor has acquired defects in MHC class I presentation. An important challenge will be to develop technologies that can deliver comprehensive tumor “immunomics” in a timely and cost-effective fashion. The aim is to provide clinicians with robust biomarkers to guide therapeutic decision making especially when it comes to the wide repertoire of possible combination therapies. An additional challenge is to take into account both the spatial and the temporal heterogeneity of a tumor for a given patient, i.e., are different metastatic sites sensitive/resistant to immunotherapy to the same extent, and how does this evolve over time during the course of specific treatments? As the field of cancer immunology further evolves, several additional questions are raised: what is the role of CD4+ T-cells in vaccine-induced anti-tumor responses? Which could be the optimal chemotherapy or radiotherapy regimen in combination with a cancer vaccine? Does the gut microbiome impact on cancer vaccine efficacy the same way as it influences responses to checkpoint inhibitors? As difficult as these challenges may be, the reward is considerable given the excellent tolerability of vaccines and the promise of long term protective immunological memory, which may transform disease control into cure.

## Author Contributions

The author confirms being the sole contributor of this work and has approved it for publication.

### Conflict of Interest Statement

The author declares that the research was conducted in the absence of any commercial or financial relationships that could be construed as a potential conflict of interest.

## Abbreviations

ASC: Apoptosis-Associated Speck-Like Protein Containing CARD
CCL: CC chemokine ligand
cGAS: Cyclic GMP-AMP synthase
CSF-1R: Colony-stimulating factor receptor-1
CTLA-4: Cytotoxic T-Lymphocyte Associated Protein 4
IFN: Interferon
IKK: IκB kinase
IL: Interleukin
IRF3: Interferon regulatory factor 3
ISCOM: Immune stimulating complexes
LMP-2: Epstein–Barr virus (EBV) latent membrane protein 2
NFκB: Nuclear Factor kappa-light-chain-enhancer of activated B cells
TAA: Tumor-associated antigen
TAP-1, Transporter 1: ATP Binding Cassette Subfamily B Member
TBK1: TANK Binding Kinase 1
TCR: T-cell receptor
TGF-β: Transforming growth factor beta
TRAIL: TNF-related apoptosis-inducing ligand.
